# Magnitude of Neonatal Hypothermia and Its Risk Factors Among Hospitalized Neonates in Southern Ethiopia

**DOI:** 10.1155/ijpe/4337114

**Published:** 2025-08-04

**Authors:** Anteneh Gashaw, Hunduman Bedada, Eyob Abera

**Affiliations:** Department of Midwifery, College of Medicine & Health Sciences, Dilla University, Dilla, Ethiopia

**Keywords:** Dilla, Ethiopia, intensive care unit, neonatal hypothermia

## Abstract

**Background:** Newborn hypothermia is a critical global health challenge, particularly in low-resource settings, where it significantly contributes to neonatal morbidity and mortality. A mere one-degree drop in a newborn's body temperature can substantially increase the risk of death. Understanding hypothermia factors is key to developing strategies to reduce neonatal mortality. Despite its status as a leading cause of neonatal death, no studies have been conducted in the study area to determine the prevalence and associated factors of neonatal hypothermia.

**Method:** An institution-based cross-sectional study of 237 participants employed systematic random sampling. Data collection involved interviews and chart reviews, analyzed using SPSS Version 27.0. Bivariable logistic regression identified associations with a *p* value < 0.25, and multivariate logistic regression determined significant factors with a *p* value < 0.05.

**Result:** The prevalence of neonatal hypothermia among newborns in this study was 54% (128 cases). Mothers whose labor was induced were 2.3 times more likely to have a hypothermic newborn (AOR = 2.276, 95% CI: 1.019–5.081). Newborns delivered at home were seven times more likely to develop hypothermia (AOR = 7.031, 95% CI: 1.018–48.582). Additionally, mothers without pregnancy complications were 0.4 times less likely to have a hypothermic baby compared to those who experienced pregnancy complications (AOR = 0.464, 95% CI: 0.235–0.997).

**Conclusion:** The prevalence of neonatal hypothermia in the study area was found to be 54%. Factors associated with neonatal hypothermia included labor induction, home delivery, and complications during pregnancy.

## 1. Introduction

The World Health Organization defines hypothermia as a core body temperature below 36.5°C (97.7°F) [1, 2]. It is classified into three levels of severity: mild or cold stress (36.0°C–36.4°C), moderate (32.0°C–35.9°C), and severe (below 32°C) [3].

Neonatal hypothermia is a widespread issue globally, even in hot tropical regions [4]. Its prevalence varies between 11% and 95% worldwide [1, 4–6]. In developing countries, hypothermia is a significant contributor to neonatal mortality and morbidity, increasing the risk of death fivefold [1, 4]. Recent studies indicate that a 1°C drop in body temperature raises mortality risk by 80% [1, 7].

The first few minutes after birth are critical for an infant's survival, marking the transition from intrauterine to extrauterine life with various physiological adaptations [4]. A sudden drop in ambient temperature during delivery, particularly without proper preventive measures, can result in neonatal hypothermia [2, 8]. This condition may arise purely from environmental factors or indicate an underlying illness, such as sepsis [7, 9]. Ensuring an optimal environmental temperature in the delivery or operating room is essential to prevent neonatal hypothermia [9]. In preterm infants, hypothermia significantly raises the risk of morbidity and mortality [2, 8].

Bathing newborns within the first day of life, low socioeconomic status, inadequate kangaroo mother care practices, delayed initiation of breastfeeding beyond the first hour, traditional oil massage of neonates, and limited knowledge of thermal care among healthcare providers have been identified as key factors contributing to neonatal hypothermia [3, 5, 6]. In Ethiopia, the prevalence of neonatal hypothermia is notably high, ranging from 50% to 70% [5, 6, 10]. Studies conducted both internationally and in Ethiopia have identified various factors associated with neonatal hypothermia [6, 10]. These include poverty, home delivery, absence of skin-to-skin contact, low birth weight, prematurity, early bathing, delayed breastfeeding initiation, traditional oil massage practices, and insufficient knowledge of thermal care among health workers [6, 11].

Although neonatal hypothermia is recognized as a major contributor to neonatal mortality worldwide, there is a lack of research in the study area specifically addressing its prevalence and associated factors. This gap in evidence is particularly concerning given the critical importance of understanding and addressing the determinants of neonatal hypothermia to develop effective prevention and management strategies tailored to the local context. Without such data, it remains challenging to design targeted interventions to reduce neonatal mortality and improve overall newborn health outcomes in the region.

## 2. Method

### 2.1. Study Design, Area, and Period

An institutional based cross-sectional study design was conducted in the Neonatal Intensive Care Unit (NICU) of Dilla University Teaching Hospital (DUTH) in Dilla Town, Gedeo Zone, from September to October 2024. The hospital is located in southern Ethiopia, approximately 359 km south of Addis Ababa and 90 km from Hawassa, the capital city of Ethiopia and Southern regional state of Ethiopia. Initially established as a district hospital in 1977 E.C.

Currently, DUTH provides preventive, curative, and rehabilitative services to a catchment population of about two million people across various wards. The NICU is staffed with trained nurses, general practitioners, and pediatricians and is equipped with essential resources, including oxygen sources, mechanical ventilators, CPAP machines, warmers, and phototherapy units. The hospital employs 1341 staff members, of whom 686 work in administration and 261 are healthcare professionals.

### 2.2. Population

The source population for this study included all neonates admitted to the NICU at DUTH, while the study population consisted of selected neonates admitted to the NICU and present during the data collection period.

### 2.3. Eligibility Criteria

The inclusion criteria for this study encompassed all neonates admitted to the NICUs of DUTH along with their mothers during the study period. Exclusion criteria included neonates whose mothers were not present during the study period, those without medical records at admission, and neonates whose mothers were unconscious during the study period.

### 2.4. Sample Size Determination

The sample size for this study was calculated by using a single population proportion formula by considering the following assumption.

 $$\begin{array}{c} n=\frac{\left(\text{Za}/2\right)2*p\left(1-p\right)}{\left(d\right)2}, \end{array}$$

where *P* were the prevalence of neonatal hypothermia from the study conducted in Addis Ababa public hospital which is 83.17% [6], *n* is the required sample size (minimum sample size), *Z* is the critical value at 95% CI which is equal to 1.96, *P* is the prevalence rate of hypothermia (83.17), and *d* is the margin of error tolerated (level of significance) (5% *d* = 0.05).

$$\begin{array}{cc} \; & n=\frac{\left(1.96\right)2*0.8317\left(1-0.8371\right)}{\left(0.05\right)2}=\frac{3.8416*0.8317*0.1683}{0.0025},\\ n=215. \end{array}$$

After adding a 10% nonrespondent rate, the final sample size was 237.

### 2.5. Sampling Technique and Procedure

First, the average number of neonatal admissions over 6 months at DUTH was reviewed using monthly reports. This provided an estimated average of 480 neonatal admissions over 2 months. Subsequently, the sampling interval (*k*) was calculated for systematic random sampling, resulting in a *k* value of 2. Therefore, every second neonatal admission was selected for the study.

### 2.6. Data Collection Methods

Data on maternal and neonatal risk factors associated with neonatal hypothermia were collected using a structured, interviewer-administered questionnaire and a chart review. The tool was adapted by reviewing relevant literature and initially prepared in English. It was then translated into Amharic and the local language, Gede'uffa, before being back-translated into English to ensure consistency and clarity. Data collection took place between September and October 2024 GC.

Two experienced nurses served as data collectors, and one supervisor with prior experience in overseeing data collection was recruited. The collected data were reviewed and checked for completeness prior to entry. Information was gathered at the time of the neonate's admission to the NICU and supplemented by reviewing records from registration books in the labor ward, NICU, and gynecology ward.

### 2.7. Variables

The dependent variable in this study was neonatal hypothermia, while the independent variables included various sociodemographic, neonatal, obstetrical, and environmental factors. Sociodemographic factors encompassed maternal age, educational status, occupation, and residence. Neonatal factors included the baby's age, sex, birth weight, whether the baby's head was covered with a cap at birth, medical diagnosis at admission, and whether the baby was bathed within 24 h of birth. Obstetrical and environmental factors covered the onset of labor, parity, distance to the nearest health facility, time and place of delivery, mode of delivery, NICU room temperature, and obstetric complications during pregnancy.

### 2.8. Operational Definition

Hypothermia: an axillary temperature of less than 36.5°C.

Admission temperature: The first temperature obtained from neonates at admission to NICU [6].

Inborn refers to a newborn delivered at the study hospital, while outborn refers to a newborn delivered at a location other than the study hospital [6].

Neonatal hypothermia is classified as mild when the axillary temperature ranges from 36.0°C to 36.4°C, moderate when it ranges from 32.0°C to 35.9°C, and severe when it is less than 32.0°C [6].

### 2.9. Data Collection Procedure and Quality Assurance

Data were collected using Kobo Collect and structured interviewer-administered questionnaires, adapted from previous studies and supplemented by chart reviews. The questionnaire included sections on the mother's sociodemographic characteristics, obstetric and environmental factors, and neonatal-related variables. It was originally prepared in English and translated into Amharic and Gede'uffa to ensure consistency and clarity. The tool was pretested on 5% of the sample size at Gedeb Primary Hospital, located in Gedeo Zone.

To ensure data quality, the questionnaire was carefully designed, interviewers were thoroughly trained in data collection procedures, and proper categorization and coding of the questionnaire were implemented. Each day, data collectors reviewed and checked the questionnaires for completeness the following morning before continuing data collection. The collected data were also reviewed for completeness prior to entry.

### 2.10. Data Analysis

After data collection, all completed questionnaires were reviewed for completeness, consistency, and accuracy. The data were then entered and analyzed using SPSS (Statistical Package for Social Sciences) Version 27. Descriptive statistics, such as frequency tables and pie charts, were used to summarize the data. To evaluate the association between independent variables and the outcome variable, bivariable logistic regression was performed. Variables with a *p* value less than 0.25 in the bivariable analysis were further examined using multivariable logistic regression. The strength of associations was measured using odds ratios (ORs) with 95% confidence intervals (CIs). Variables with a *p* value of less than 0.05 in the multivariable analysis were considered significantly associated with the outcome variable.

## 3. Result

### 3.1. Sociodemographic Characteristic

A total of 237 mothers and their neonates participated in the study, achieving a 100% response rate. The mean age of the mothers was 29 years, with the largest proportion (31.5%) falling within the age group of 25–29 years. The majority of mothers, 102 (43%), identified as Protestant, and 153 (64.6%) were urban residents. Regarding education, 113 (47.7%) had completed secondary education, and 102 (43%) were housewives. Additionally, 123 (51.9%) of the mothers were multiparous, and 136 (57.4%) traveled more than 10 km from their homes to the nearest health facility (Table 1).

### 3.2. Obstetric-Related Factors

Eighty-five (35.9%) mothers had obstetric complications during pregnancy. More than half of the pregnancies, 230 (97%) were singleton pregnancies, and the majority of neonates 152(64.1%) were born without any obstetric complications. More than half 139(59.6%) were delivered through SVD. 115(48.5) had no skin-to-skin contact immediately after birth. Around 136 (57.4%) were multiparous mothers (Table 2).

### 3.3. Prevalence of Hypothermia

**Figure 1:**
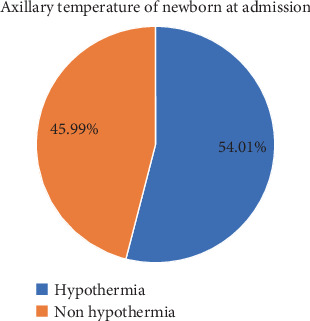
Prevalence of neonatal hypothermia among new born admitted to neonatal intensive care unit of Dilla University Teaching Hospital, Southern Ethiopia (2024).

The prevalence of neonatal hypothermia in DUTH among neonates admitted to NICU was 128 in number, which was 54% with 95% CI: (47.6%–0.60.3%) (Figure 1). A total of 79 neonates (61.71%) had mild hypothermia, 40 neonates (31.25%) had moderate hypothermia, and the remaining 9 neonates (7.03%) were classified as having severe hypothermia.

### 3.4. Neonatal-Related Factors

The majority of the neonates were male, accounting for 143 (60.3%) of the total. Most of the neonates, 132 (55.7%), were older than 24 h at the time of data collection. Over half, 145 (61.8%), had a birth weight of 2500 g or more. Similarly, 146 (61.8%) of the neonates were born at a gestational age of more than 37 weeks. Among those, 48 neonates developed hypothermia, which accounts for around 37.5%. About 115 (48.5%) did not begin breastfeeding within the first hour after birth. Additionally, 154 (65%) required resuscitation at birth. Medical records showed that 54 (22.8%) of the neonates were diagnosed with sepsis (Table 3).

### 3.5. Environmental-Related Factors

Twenty-nine (12.2%) of the newborns were bathed within the first 24 h after birth. Seventy (29.5%) were outborn neonates, with 24 (10.1%) delivered in private health facilities. More than half, 160 (67.5%), were born during the daytime. The majority of the neonates, 221 (93.2%), were admitted to the NICU at a room temperature of 25°C or higher (Table 4).

### 3.6. Factors Associated With Neonatal Hypothermia

In bivariate logistic regression analysis, factors significantly associated with hypothermia were age of the mother, sex of new born, gestational age, provision of skin-to-skin contact, complications during pregnancy, clinical diagnoses during admission, onset of labor, delivery in an outborn setting. Variables revealed as significant on bivariate analysis were introduced into multiple logistic regression. In multiple logistic regression analysis, factors that were significantly associated with hypothermia were complication during pregnancy, onset of labor, and deliver setting.

Mothers whose labor was induced were 2.3 times more likely to have a hypothermic newborn compared to those whose labor began spontaneously (AOR = 2.276, 95% CI: 1.019–5.081). Newborns delivered at home were seven times more likely to develop hypothermia compared to those born in a hospital setting (AOR = 7.031, 95% CI: 1.018–48.582). Additionally, mothers without pregnancy complications were 0.4 times less likely to have a hypothermic baby compared to those who experienced pregnancy complications (AOR = 0.484, 95% CI: 0.235–0.997) (Table 5).

## 4. Discussion

The prevalence of neonatal hypothermia in the study area was 128 (54%) with a 95% CI of 47.6%–60.3%. This finding is closely aligned with findings from similar studies in Zambia (57%), Southwest Ethiopia (50.3%), Pakistan (49.5%), Iran (47.8%), East Africa (57.2%), and Jinka General Hospital (58.6%) [5, 12–14]. This similarity is likely due to the consistent definition of neonatal hypothermia, comparable research methodologies, similar levels of awareness and education, and common risk factors across these regions. This rate is lower compared to studies conducted in Bahir Dar, Ethiopia (67%) [5], Gondar, Northwest Ethiopia (69.8%) [15], and Addis Abeba 64% [6]. These differences may be attributed to variations in sample sizes or the specific populations studied, such as different age groups, geographic locations, or risk groups. Larger or more at-risk populations tend to show higher prevalence rates. Additionally, the criteria for defining hypothermia can differ, with some studies applying stricter thresholds for core body temperature, while others include borderline or varying degrees of hypothermia. The type of study design (cross-sectional, cohort, or case-control) can also impact prevalence rates. Seasonal or geographical variations, which affect exposure to cold, may further influence the occurrence of hypothermia. Lastly, studies based on hospital records might overlook milder cases that do not require medical attention.

In this study, the prevalence of neonatal hypothermia is relatively high compared to a study conducted in Addis Ababa public hospitals, which reported a prevalence of 19.3% [13]. This difference may be attributed to a sample size difference, variations in methods for measuring body temperature, and differences in population characteristics.

One of the factors associated with neonatal hypothermia in this study was the presence or absence of complications during pregnancy. Mothers without pregnancy complications were 0.4 times less likely to have a hypothermic baby compared to those with complications. This finding aligns with a retrospective cohort study examining the influence of maternal and perinatal complications on therapeutic hypothermia in newborns [16]. Complications such as gestational diabetes, hypertension, or placental insufficiency can alter the intrauterine environment. Insufficient nutrients and oxygen supply to the fetus may result in lower body fat stores, essential for thermoregulation and heat retention. Many pregnancy complications can lead to preterm birth, and preterm infants are especially prone to hypothermia due to lower body fat and immature thermoregulatory systems. They may also lack adequate brown fat, vital for heat production [14, 17]. Additionally, complications can lead to low birth weight, which is linked to a higher risk of hypothermia due to a greater surface area-to-volume ratio, resulting in increased heat loss [14].

Another factor associated with neonatal hypothermia was the onset of labor. Mothers whose labor began with induction were 2.3 times more likely to have a hypothermic newborn compared to those whose labor started spontaneously. This finding is supported by a multicenter study [14, 17]. If induction occurs before the fetus is fully mature, the baby may be born with lower body fat and immature thermoregulation capabilities, increasing the risk of hypothermia. Induced labor can also be more stressful for the fetus than spontaneous labor [17]. The use of labor-inducing medications, such as oxytocin, can accelerate labor progression, potentially leading to fetal distress. This stress may affect the newborn's ability to regulate body temperature after birth. Additionally, induced labor may increase the likelihood of interventions like cesarean sections, especially if complications arise. Newborns delivered via cesarean may experience different physiological transitions than those delivered vaginally, potentially impacting their ability to regulate body temperature [18].

Another factor identified as contributing to neonatal hypothermia in this study was the delivery setting. Newborns delivered at home were 7.0 times more likely to develop hypothermia compared to those born in hospitals. This finding is supported by a prospective cohort study conducted in northern Nigeria [19]. This might be due to home deliveries often lacking essential medical equipment, such as radiant warmers or incubators, to maintain the baby's body temperature. Additionally, birth attendants at home may not be adequately trained to recognize and manage hypothermia or implement immediate warming techniques like skin-to-skin contact. Environmental factors, such as lower room temperatures, can also increase the risk of heat loss. Delays in initiating essential newborn care practices, such as drying and wrapping the baby, further contribute to the risk. Moreover, limited access to emergency care in case of complications exacerbates the likelihood of hypothermia in home-born neonates compared to those born in hospitals.

In Ethiopia, delivery and postnatal care services are required to adhere to the WHO standard operating procedures (SOPs) for essential newborn care, including hypothermia prevention. In this study, the neonates were referred from both inborn and outborn facilities within the catchment area, and all these hospitals reportedly follow a standardized protocol for hypothermia prevention. Although neonates are heat-sensitive by nature, the ambient temperature of the study area is relatively moderate, which may slightly reduce the risk of hypothermia. However, consistent implementation of the warm chain, availability of hypothermia prevention bundles, and proper temperature monitoring practices remain essential regardless of external temperature. The study setting's adherence to these protocols was considered part of routine service, but the environmental and operational factors should still be acknowledged as potential confounders in interpreting the results.

## 5. Limitation

However, due to the nature of the study design, this cross-sectional study limits the ability to establish temporal or causal relationships between the identified factors and neonatal hypothermia. In addition, it does not allow for the assessment of potential complications or outcomes resulting from neonatal hypothermia. Therefore, we recommend that future researchers consider conducting prospective cohort or case-control studies to investigate the short- and long-term complications associated with neonatal hypothermia.

## 6. Conclusion

The prevalence of Neonatal hypothermia among new born neonates admitted to NICU was high at 54%. The presence of complications during pregnancy, onset of labor, and delivery setting were significantly associated with neonatal hypothermia among new born neonates admitted to NICU. Promoting institutional delivery in the study area is a crucial strategy to reduce the incidence of neonatal hypothermia.

## Figures and Tables

**Table 1:** Sociodemographic characteristics of participants in prevalence of hypothermia and associated factors among neonates admitted to NICU of Dilla University Teaching Hospital (2024).

**Variables**	**Categories**	**Frequency**	**Percentage (%)**
Age of mother	15–19	15	6.3
	20–24	62	26.2
	25–29	75	31.6
	30–34	46	19.4
	35–39	32	13.5

Religion	Orthodox	69	29.1
	Muslim	39	16.5
	Protestant	102	43.0
	Catholic	11	4.6
	Other	16	6.8

Residence	Urban	153	64.6
	Rural	84	35.4

Occupation of the mother	House wife	102	43.0
	Government employ	38	16.0
	Private business	71	30.0
	Student	12	5.1
	Farmer	14	5.9

Educational status of the mother	Unable to read and write	14	5.9
	Primary school	53	22.4
	Secondary education	113	47.7
	Diploma and above	57	24.1

**Table 2:** Obstetric-related factors in the prevalence of hypothermia and associated factors among neonates admitted to the NICU of Dilla University Teaching Hospital (2024).

**Variables**		**Frequency**	**Percentage**
Skin to skin contact immediately after birth	Yes	122	51.5
	No	115	48.5

Obstetric complication during pregnancy	Yes	85	35.9
	No	152	64.1

Pregnancy type	Single	229	96.0
	Twine	6	2.5
	Triple	2	0.8

Mode of deliver	SVD	139	59.6
	Instrumental	14	5.9
	C/S	84	35.4

Onset of labor	Spontaneous	162	68.4
	Induced	75	31.6

Parity	Primipara	114	48.1
	Multipara	123	51.9

**Table 3:** Neonatal related factors in prevalence of hypothermia and associated factors among neonate admitted to NICU of Dilla University Teaching Hospital (2024).

**Variable**		**Frequency**	**Percentage (%)**
Age of neonate	< 24 h	105	44.3
	> 24 h	132	55.7

Sex of neonate	Male	143	60.3
	Female	94	39.7

Birth weight	< 2500 g	92	38.5
	> 2500g	110	46.4
	≥ 400 g	35	14.7

Neonatal Age at birth	< 37 weeks	91	38.4
	> 37 weeks	146	61.6

Receive resuscitation at birth	Yes	83	35
	Non	154	65

Breast feeding within 1 h of delivery	Yes	122	51.5
	No	115	48.5

Clinical diagnosis during admission	Asphyxia	16	6.8
	Congenital anomaly	6	2.5
	Hypoglycemia	31	13.1
	Jaundice	18	7.6
	LBW	28	11.8
	MAS	12	5.1
	Preterm	47	19.8
	Respiratory	25	10.5
	Sepsis	54	22.8

**Table 4:** Environmental related factors in prevalence of hypothermia and associated factors among neonate admitted to NICU of Dilla University Teaching Hospital (2024).

**Variable**		**Frequency**	**Percentage**
Bathed your baby before 24 h old	Yes	29	12.2
	No	208	87.8

Where did you delivery	In born	167	70.5
	Outborn	70	29.5

Setting for outborn delivery	Other hospital	20	28.6
	Health center	19	27.12
	Private health facility	12	17.16
	Home	19	27.12

Time of delivery	Day time	160	67.5
	Night time	77	32.5

Room temp of NICU	≥ 25°C	221	93.2
	< 25°C	16	6.8

**Table 5:** Factors associated with neonatal hypothermia among neonates admitted to the NICU of Dilla University Teaching Hospital (2024).

**Variables**	**Category**	**Hypothermia**		**COR (95% CI)**	**AOR (95% CI)**
		**Yes**	**No**		
Had complication during pregnancy	Yes	43	42	1	1
	No	85	67	0.239 (0.128–2.110)	0.484 (0.235–0.997)⁣^∗^

Onset of labor	Spontaneous	96	66	1	1
	Induced	32	43	1.955 (1.122–3.403)	2.276 (1.019–5.081)⁣^∗^

Delivery setting	Hospital	7	13	1	
	Health center	12	7	2.315 (0.960–5.582)	
	Private hospital	3	9	0.810 (0.304–2.159)	
	Home	12	7	2.579 (0.980–6.787)	7.031 (1.018–8.582)⁣^∗^

Abbreviations: AOR, adjusted odds ratio; CI, confidence interval; COR, crude odds ratio.

⁣^∗^*p* < 0.05.

## Data Availability

The datasets generated and/or analyzed during the current study are not publicly available due to preserving participant anonymity but are available from the corresponding author on reasonable request (Anteneh Gashaw: antenehgashaw77@gmail.com).

## Nomenclature

AOR: adjusted odds ratio
CI: confidence interval
COR: crude odds ratio
PNC: postnatal care
SNNPR: South Nations, Nationalities, and Peoples' Region
